# Influence of planting methods and organic amendments on rice yield and bacterial communities in the rhizosphere soil

**DOI:** 10.3389/fmicb.2022.918986

**Published:** 2022-07-28

**Authors:** Zhiqiang Tang, Liying Zhang, Na He, Zhiqi Liu, Zuobin Ma, Liang Fu, Hui Wang, Changhua Wang, Guomin Sui, Wenjing Zheng

**Affiliations:** ^1^Liaoning Rice Research Institute, Shenyang, China; ^2^Liaoning Academy of Agricultural Sciences, Shenyang, China

**Keywords:** paddy rice, biochar, high-throughput 16S rRNA sequencing, bacterial communities, enzyme activities

## Abstract

A comprehensive understanding of rice cultivation techniques and organic amendments affecting soil quality, enzyme activities and bacterial community structure is crucial. We investigated two planting methods (direct seeding and transplanting) of paddy rice (Oryza sativa) and organic amendments with rice straw and biochar on crop yield and soil biological and physicochemical properties. Rhizosphere bacterial communities at the maturity stage of rice growth were characterized through high-throughput 16S rRNA sequencing. Soil biochemical properties and enzyme activity levels were analyzed. Grain yield of paddy rice with transplanting increased 10.6% more than that with direct seeding. The application of rice straw increased grain yield by 7.1 and 8.2%, more than with biochar and the control, respectively. Compared to biochar and the control, the application of rice straw significantly increased sucrase, cellulase, protease, organic carbon, available phosphorus, nitrate, and ammonium. The application of biochar increased microbial biomass nitrogen and carbon, urease, pH, available nitrogen, and available potassium compared to the application of rice straw and the control. Principal coordinate analysis and dissimilarity distances confirmed significant differences among the microbial communities associated with planting methods and organic amendments. *Bacteroidetes*, *Nitrospirae*, *Firmicutes*, and *Gemmatimonadetes* abundance increased with rice straw relative to biochar and the control. The biochar addition was associated with significant increases in *Chloroflexi*, *Patescibacteria*, *Proteobacteria*, and *Actinobacteria* abundance. Pearson’s correlation analyzes showed that *Chloroflexi*, *Bacteroidetes* and *Nitrospirae* abundance was positively correlated with grain yield. The relative abundance of these bacteria in soil may be beneficial for improving grain yield. These results suggest that planting methods and organic amendments impact soil biochemical characteristics, enzyme activity levels, and microbial community composition.

## Introduction

Rice production has a long history in China, and results in large volumes of crop residue byproducts (Liu et al., 2008). The two primary approaches to producing paddy rice (*Oryza sativa*) production are direct seeding and mechanized transplantation. Transplanting is a method to establish rice after growing seedlings in the nursery (Susilawati et al., 2021), and the field needs to go through the process of soaking, raking and settling. Transplanting is labor intensive and financially expensive. Dry direct seeding is a simple approach to rice cultivation in which rice seeds are sown on dry land, after that, a water layer is established following seedling emergence and development to the 3-leaf stage. Direct seeding obviates the need for seedling nurseries, seeding management, or transplanting, thereby improving overall mechanical efficiency and lowering labor costs (Ashraf et al., 2017a,b). Planting methods alter tilling systems in the field, which will affect the root growth environment and may indirectly affect the rhizosphere microbial community structure.

In Northeastern China, a common post-harvest approach to disposing of rice straw entails burning these crop residues after the annual harvest and before planting during the following spring. However, this process leads to the waste of potentially valuable crop resources, induces severe air pollution, and can accelerate the loss of organic matter from the underlying soil (Cao et al., 2008). Thus, more environmental friendly and efficient approaches to utilizing these natural resources are urgently needed. One strategy aimed at remediating this issue is the return of crop straw to the field, which can protect from soil degradation, thereby simultaneously reducing resource waste while maintaining soil fertility in intensive cultivation systems (Sun et al., 2015). Another approach relies on the preparing of rice straw-derived biochar that can then be applied to fields, thereby modulating soil quality characteristics in a manner beneficial to overall soil productivity, nutrient availability, and soil health (Novak et al., 2014). Biochar application can also reportedly improve the pH of acidic soils and reduce nutrient losses through the enhancement of soil water retention (Joseph et al., 2010).

Soil microbes are essential regulators of soil health, crop productivity, and sustainability of cultivated plants (Berendsen et al., 2012). The rhizosphere is the interface between plant roots and soil that is affected by environmental factors, microbial richness and diversity (Phillips et al., 2013). Recent studies have shown that plant roots selectively recruit specific root microbial communities from the soil, which impact crop growth, development, and grain yield (Edwards et al., 2015; Chen et al., 2019). Soil microorganisms and plants live in the same environment, interact and influence each other; many of which help crops obtain nutrients from the soil, promote the maintenance of soil fertility, and make crops grow healthily, and ultimately affect grain yield formation. Therefore, crops usually utilize rhizosphere microorganisms and roots for nutrient-absorbing, growth-promoting, and grain yield-improving (Fierer, 2017; Zhong et al., 2020; Wang et al., 2021). Therefore, understanding the relationship between crops and rhizosphere-related microbial communities is of great agronomic significance for improving agricultural productivity and sustainable development.

The straw application represents a practical approach to improving soil quality through increases in enzymatic activity and regulating soil microbial activity (Bastida et al., 2007). The straw application can influence the composition and diversity of soil microbial communities (Qin et al., 2015; Sun et al., 2016). Biochar has a high organic carbon content, which increases the soil carbon content after application to the soil and affects the community and diversity of microorganisms (Steinbeiss et al., 2009; Cayuela et al., 2013). As biochar is highly porous, it can serve as a favorable habitat in which many microorganisms can thrive and proliferate (Jia et al., 2017).

Soil enzymes are biologically active substances with specific catalytic functions involved in energy flow and the recycling of mineral elements in soil ecosystems. Soil enzyme activity is an essential indicator linking soil microbial community and soil nutrient availability (Nannipieri et al., 2017). Soil enzymes are one of the factors that reflect the activity of soil microorganisms, which can directly and indirectly reflect soil nutrient conditions and serve as an essential indicator for evaluating soil nutrient availability (Cotrufo et al., 2015).

Establishing the linkage between soil bacteria, enzyme activities and soil nutrients has been a long-standing and complex topic in soil ecology. We attempted to tackle this question with specific objectives: (1) to evaluate the impact of planting methods and organic amendments on the grain yield of paddy rice, (2) to determine the response pattern of rhizospere soil microbial taxonomy, microbial composition and soil biochemical properties and enzymes, and (3) to identify differences and similarities in bacteria communities as impacted by planting methods and organic amendments.

## Materials and methods

### Experimental design

This study was conducted at the Liaoning Rice Research Institute (40°57′ N, 122°14′ W, altitude: 41.5 m) in Shenyang, Liaoning Province, China. The field experiment was carried out in 2019. The study site is located in a region with a semi-humid temperate and monsoonal climate, an annual average temperature of 8.3°C, and average yearly precipitation of 545 mm. This experiment was conducted using a split-plot, fully randomized complete block design with three replicates. Experimental treatments included paddy rice seeding methods [direct seeding (DS) and transplanting (TP)] and organic amendments [rice straw (RS) applied at 9750 kg ha^–1^, biochar (BC) applied at 3450 kg ha^–1^, and a control (CK) without rice straw or biochar amendment]. Application rate of RS is based on average amount of rice straw produced in the region. The soil at the study site was clay loam and contained 18.6 g kg^–1^ organic matter, 1.31 g kg^–1^ total N, 1.24 g kg^–1^ total P, 24.6 g kg^–1^ total K, 108.6 mg kg^–1^ available N, 23.8 mg kg^–1^ available P, 46.2 mg kg^–1^ available K, and had a pH (H_2_O) of 5.3 at the start of the study period. The ‘Liaoxing 21′ rice cultivar was used. This cultivar was selected as it was commonly grown by local farmers in the northern region of Liaoning Province. Rice straw was prepared by chopping dry straw into 20-50 mm pieces, yielding RS containing 35% C and 0.7% N. Biochar was manufactured via the pyrolysis of prepared RS under low-oxygen conditions at 450°C for 1 h (Jin and Fu Agriculture Development Co., Ltd.) (Sui et al., 2016). This approach converted approximately 35% of the RS into small granular (diameter: 2-5 mm) particles. The prepared BC contained 66% C and 0.8% N, and had a pH of 8.7 (1:2.5 H_2_O). Each plot was 240 m^2^ in size. RS and BC were applied to appropriate experimental plots in April 2019 before rice planting. These organic amendments were incorporated into the soil by hand with a rake, and all plots were mechanically plowed to a 200-mm depth. Compound fertilizers were applied as a basal fertilizer on the soil surface and mechanically incorporated into the 0–100-mm layer by raking. The soil was additionally amended with compound fertilizers (750 kg ha^–1^, containing 12% N, 12% P, and 12% K), with additional urea applied at the seedling establishment (270 kg ha^–1^) and at the mid-tillering stage of rice growth (270 kg ha^–1^). For the TP, rice seedlings were sown in a nursery bed on April 19, 2019, and were transplanted into paddy fields on May 26, 2019. For the DS, rice seeds were mechanically planted on April 27, 2019 under dry conditions (5.5 million seeds ha^–1^). All paddies were harvested on October 20, 2019. The same amount of N, P, and K was used for paddy rice with the TP and DS. Water levels, weed growth, insects, and diseases were managed as necessary to minimize yield losses.

### Rhizosphere soil collection

On October 2, 2019, five hills of rice were selected from each plot, and soil samples with an area of 200 × 200 mm in a depth of 200 mm were excavated for each hill. Each hill consisted of approximately 15 plants. Briefly, five soil core samples from each plot were pooled together to generate a composite sample. Excessive soil was removed by shaking the roots of the plant until the roots still having soil attached. The roots were separated from the soil by vigorously shaking the root system with sterile forceps. The soil samples separated from the roots were then placed in a 500 mL sterile flask. Following vigorously stirring of this mixture using sterile forceps, the collected rhizosphere soil was separated into two aliquots, with one being frozen at –80°C in a 50 ml tube for subsequent DNA extraction, whereas the other was stored at 4°C to measure enzyme activity levels, microbial biomass, and soil physicochemical properties.

### Soil enzyme activity and microbial biomass nitrogen and carbon analyzes

The activity of soil enzymes was measured using the method of Guan et al. (1986). Soil weight for determination of enzyme activity was 5 g. Soil saccharase activity was measured using the 3,5-dinitrosalicylic acid colorimetric method. The incubation was carried out at 37 °C for 24 h. The amount of glucose produced per gram of soil per day was defined as the unit of saccharase activity. Urease activity was determined by sodium phenol colorimetry. The incubation was carried out at 37 °C for 24 h. The production of 1 μg of ammonia nitrogen per gram of soil sample per day was defined as the unit of urease activity. Cellulase activity was determined using carboxymethy cellulose as a substrate and glucose as a product after incubation at 37°C for 72 h. Protease activity was determined by the ninhydrin colorimetric method. The incubation was carried out at 30°C for 24 h. The production of 1 mg of tyrosine per gram of soil per day was defined as the unit of protease activity. A chloroform fumigation-K_2_SO_4_ extraction approach was used to measure microbial biomass carbon (MBC) and nitrogen (MBN) (Brookes et al., 1985; Vance et al., 1987). The weight of soil used to determine the microbial biomass was 20 g. Briefly, these samples were fumigated with chloroform steam in a vacuum desiccator for 24 h, and residual chloroform was removed by repeated vacuuming. After adding 0.5 M K_2_SO_4_ or NaHCO_3_, respectively, the mixture was shaken for 30 min and filtered immediately. The filtered extract was used to measure MBC and MBN. The organic carbon and total nitrogen were used to calculate MBC and MBN. The conversion factor for MBC and MBN was 0.45 (Zang et al., 2015).

### Physicochemical property analyzes

The pH of soil samples suspended in water at a 1:2.5 soil:water ratio (*w*/*w*) was determined with a pH meter. Soil available N (AN) was quantified via a NaOH diffusion approach (Lu, 2000). Two grams of soil samples were weighed and evenly distributed into the outer chamber of the diffusion dish, and 2 ml of the boric acid indicator was added to the inner chamber. 10mol/l sodium hydroxide solution was added to the soil for alkaline hydrolysis and diffusion at 40°C for 24 h. Soil available P (AP) was extracted using NaHCO_3_, and P concentrations in filtrate samples were measured via a molybdenum blue colorimetric method (Page et al., 1982). Soil available K (AK) was extracted using ammonium acetate at a pH of 7.0 and measured via flame photometry. Soil organic carbon was quantified with a K_2_Cr_2_O_7_-H_2_SO_4_ wet oxidation method (Vos et al., 2007).

### 16s rRNA sequencing and analysis

A Fast DNA SPIN Kit for Soil (Q-BIOgene, CA, United States) was used to isolate total DNA from 0.5 g soil samples based on provided directions, after which the quantity and quality of the isolated DNA were assessed with an automated microplate reader (Synergy HTX, Gene Company Ltd.). The bacterial 16S rRNA V3-V4 region was amplified using the 338F (5′-ACTCCTACGGGAG GCAGCA-3′) and 806R (5′-GGACTACHVGGGTWTCTAAT-3′) primers (Cui et al., 2019). PCR amplification was performed in a total reaction volume of 10 μl containing DNA template (5–50 ng), Vn F (10 μM) 0.3 μl, Vn R (10 μM) 0.3 μl, KOD FX Neo Buffer 5 μl, dNTP (2 mM) 2 μl, KOD FX Neo 0.2 μl, ddH_2_O up to 10 μl (Yuan et al., 2019). After initial denaturation at 95°C for 5 min, samples underwent 25 cycles of denaturation at 95°C for 30 s, annealing at 50°C for 30 s, and extension at 72°C for 40 s, with a final incubation at 72°C for 7 min. PCR amplicons were purified with Agencourt AMPure XP Beads (Beckman Coulter, Indianapolis, IN, United States). Finally, these PCR products were sent to Beijing Biomarker Technologies Co., Ltd. for sequencing.

FLASH was used to merge the paired-end read sequencing data produced with an Illumina MiSeq 2500 instrument (Magoč and Salzberg, 2011). The combined tags were compared with the primer sequences, and those tags with more than six mismatches were discarded to filter out low-quality tags, followed by mass filtering (Bolger et al., 2014). High-quality tagged sequences were obtained by eliminating chimeric sequences (Edgar et al., 2011), with the remaining sequences clustered at a 97% similarity level (Edgar, 2013), using an OTU filtering threshold of 0.005% (Schloss et al., 2009), yielding the final effective data (effective tags). The raw FASTQ files obtained in the study for the sequencing libraries have been deposited into NCBI Sequence Read Archive (SRA) under the BioProject accession number PRJNA833904.

### Statistical analyzes

The SAS MIXED procedure was used for data analyzes (Littell et al., 2006). A split-plot design was utilized for these analyzes, with seeding methods as the main plot and organic amendments as a subplot. Data were tested for normality (Shapiro-Wilk normality test) and homogeneity of variance, and were transformed as appropriate to ensure that they conformed to a normal distribution. When ANOVAs were performed with significance (*P* < 0.05) least significant difference (LSD) tests were used to assess the significance of the impact of different treatment conditions on crop yields, enzyme activity levels, microbial biomass, and soil properties. Bioinformatics analyzes were performed using BMK Cloud (www.biocloud.net). Alpha diversity analysis was performed to study microbial community richness and diversity. Principal coordinate analyzes (PCoA) and analysis of similarities based on Jaccard distance dissimilarity matrices were used to visualize soil bacterial communities with the R Vegan package (Oksanen et al., 2018). Relationships between soil enzyme activities, soil physicochemical properties and bacterial communities were visualized via a redundancy analysis (RDA) (The R Development Core Team, 2015). Pearson’s correlation coefficients were used to detect significant relationships among abundant phyla, enzyme activity levels, and soil properties.

## Results

### Grain yields of rice

Significant differences in grain yield and yield components were observed when comparing planting methods and organic amendments in the present study (Table 1). Specifically, TP was associated with significantly higher grain yields relative to DS (*P* < 0.05). Grain yield of paddy rice with TP increased 10.6% more than that with DS. RS amendment increased grain yields by 7.1 and 8.2%, more than with biochar and the control, respectively.

**TABLE 1:** Effects of planting methods and organic amendments on grain yield and components.

Planting methods	Organic amendments	Number of panicles (104 ha^–1^)	Full grains per panicle	Percentage of filled grains (%)	1000 seed weight (g)	Yield (t ha^–1^)
Transplanting (TP)	RS	268.3	121.8	96.5	24.6	8.9
	BC	274.3	116.1	97.4	23.5	8.4
	CK	276.0	112.5	97.7	23.8	8.3
Direct seeding (DS)	RS	334.0	84.2	96.9	25.2	8.0
	BC	322.7	82.3	96.8	24.5	7.4
	CK	321.3	81.8	96.5	24.4	7.3
	LSD_(0.05)_	1.7	0.7			
Planting methods						
TP		272.9 b	116.8 a	97.2 a	23.9 b	8.5 a
DS		326.0 a	82.7 b	96.7 a	24.7 a	7.6 b
Organic amendments						
RS		301.2 a	103.0 a	96.7 a	24.9 a	8.5 a
BC		298.5 a	99.2 b	97.1 a	24.0 b	7.9 b
CK		298.6 a	97.1 c	97.1 a	24.1 b	7.8 b
Analysis of variance						
Planting methods (PM)		**	**	NS	**	**
Organic amendments (OA)		NS	**	NS	**	**
PM × OA		**	**	NS	NS	NS

Different letters indicate statistical significance at P < 5% within planting methods and application methods. *Significant at 0.05 level; **Significant at 0.01 level; NS, Not significant at 0.05 level. RS, rice straw; BC, biochar; CK, no RS or BC.

### Diversity of microbial communities

**FIGURE 1:**
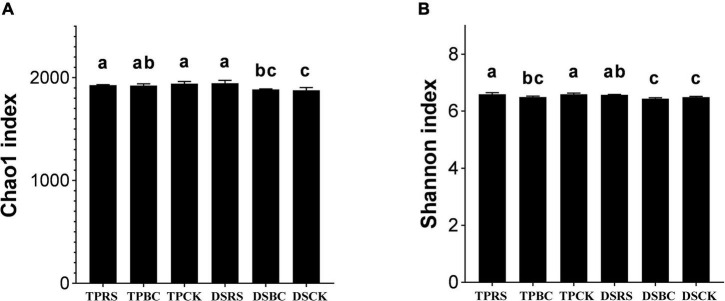
The diversity and richness of bacterial communities in the rhizosphere soil samples subjected to the planting methods and organic amendments characterized by the Chao **(A)** and Shannon indexes **(B)** (alpha diversity). Different letters indicate statistical significance at the *P* < 5% level within planting methods and organic amendments. TP, transplanting; DS, direct seeding; RS, rice straw; BC, biochar; CK, no RS or BC.

The Chao1 and Shannon indexes are shown in Figure 1. Regarding the richness of bacterial communities, the Chao 1 index showed that the bacterial richness with RS was significantly greater than that with BC and CK in DS. There was no difference in Chao 1 index among RS, BC and CK in TP. With TP, the Chao 1 index was higher than with DS. Shannon index in RS and CK treatments was significantly higher than that in the BC treatment under TP. Under DS, the Shannon index was substantially greater with RS than with BC and CK under DS. The Shannon index of TP was significantly higher than that of DS.

### Soil bacterial community structures and composition

**FIGURE 2:**
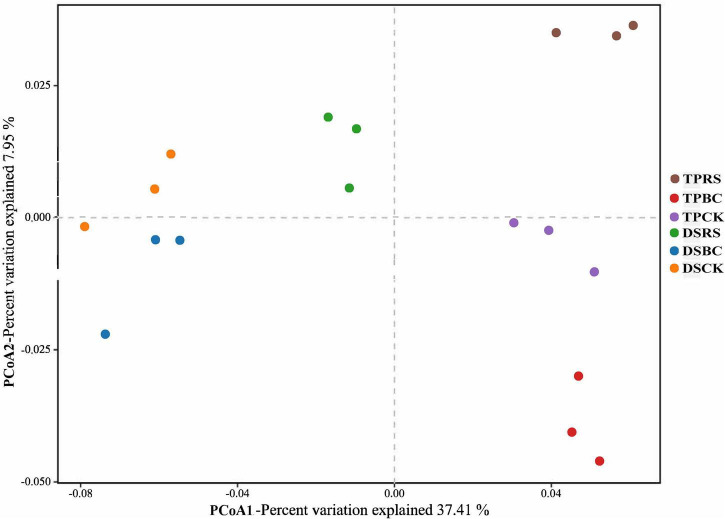
Principle coordinate analysis (PCoA) of the bacterial communities in rhizosphere soil samples. PCoA distances were based on the Jaccard distance algorithm at the OTU level. TP, transplanting; DS, direct seeding; RS, rice straw; BC, biochar; CK, no RS or BC.

Jaccard distance values were used to conduct a principal coordinate analysis at the OTU level comparing the composition of the rhizosphere microbial community as a function of planting methods and organic amendments (Figure 2). Apparent differences in microbial communities were evident among these different treatment conditions, with significant differences between the microbial communities associated with TP and DS. Moreover, within each of TP and DS, the microbial communities were distinctly separated from one another.

**FIGURE 3:**
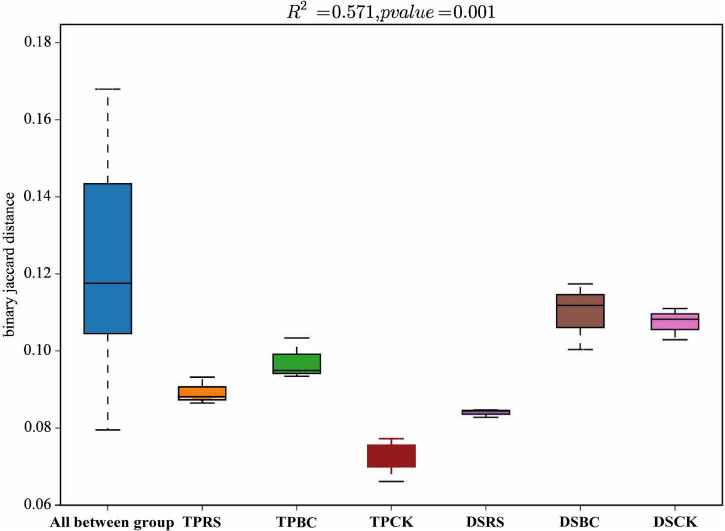
Dissimilarity distances showing the differences in bacterial communities in rhizosphere soil. Dissimilarity distances were based on the Jaccard distance algorithm at the OTU level. TP, transplanting; DS, direct seeding; RS, rice straw, BC, biochar; CK, no RS or BC.

Dissimilarity distances among these planting methods and organic amendments were further computed (Figure 3), revealing different distances in the rhizosphere soil bacterial community structures associated with TP. With DS differences in the rhizosphere bacterial communities exhibited more similar distance values with BC and CK, whereas they were distinct from the OTUs associated with the RS amendment.

**FIGURE 4:**
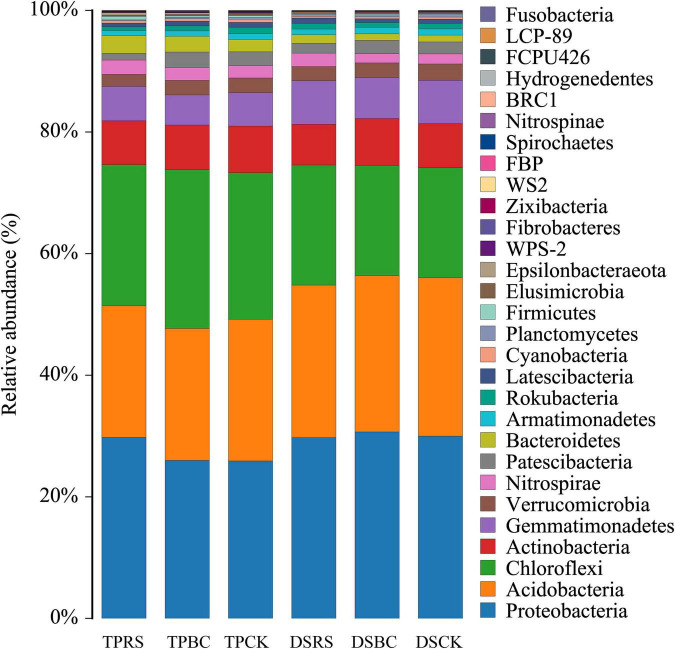
Phylum level bacterial community composition following the rhizosphere treatments. Relative abundance of different bacterial phyla in each treatments. TP, transplanting; DS, direct seeding; RS, rice straw; BC, biochar; CK, no RS or BC.

High-throughput sequencing was used to explore the composition of the rhizosphere soil bacterial communities associated with planting methods and organic amendments (Figure 4). Diverse phylum level distributions were determined for each treatment. Among the 29 phyla (Figure 4), 193 orders, 299 families and 480 genus identified in the post-processed dataset, we found that across all investigated depths, the abundant phyla (relative abundance>1%) were *Proteobacteria* (28.6%), *Acidobacteria* (23.9%), *Chloroflexi* (21.5%), *Actinobacteria* (7.3%), *Gammaproteobacteria* (6.2%), *Verrucomicrobia* (2.4%), *Nitrospirae* (2.0%), *Patescibacteria* (2.0%) and *Bacteroidetes* (1.8%), reflecting abundance patterns in planting methods and organic amendments. Their relative abundances, as well as those of all orders and genus exceeding 1% relative abundance, are shown in Supplementary Figures 1–3.

*Bacteroidetes*, *Nitrospirae*, and *Firmicutes* abundance were increased under the RS for TP relative to other treatment conditions, while *Gemmatimonadetes* abundance was significantly increased under the RS for DS. The BC addition was associated with significant increases in *Chloroflexi* and *Patescibacteria* abundance for TP and with significant increases in *Proteobacteria* and *Actinobacteria* abundance for DS. *Acidobacteria, Actinobacteria*, *Patescibacteria*, *Gemmatimonadetes*, and *Verrucomicrobia* were less abundant in the soil collected from TP relative to DS, while *Chloroflexi, Bacteroidetes, Nitrospirae*, and *Firmicutes* were more abundant in the soil collected from TP.

### Characteristics of rhizosphere soil enzyme activities and microbial biomass

Soil sucrase activity (SA), urease activity (UA), cellulose activity (CA), protease activity (PA), MBN, and MBC differed significantly among treatment conditions (Table 2). These selected enzymes were used to evaluate the level of nutrients and the decomposition of organic matter. Specifically, MBN, MBC, and CA decreased significantly for TP relative to DS, whereas SA and PA exhibited the opposite trend. RS amendment was associated with an increase in SA, CA, and PA relative to BC and CK amendment conditions, while MBN, MBC, and UA increased significantly under conditions of BC amendment relative to RS and CK amendment. RS application improved rhizosphere soil SA and PA more effectively for TP related to DS. Under conditions of DS, BC amendment was associated with higher MBN, MBC, and UA relative to RS and CK treatment conditions, while RS amendment improved rhizosphere soil CA relative to BC and CK treatment conditions.

**TABLE 2:** Soil enzyme activity and microbial biomass under different treatments of planting methods and organic amendments.

Planting methods	Organic amendments	Microbial biomass N (mg kg^–1^)	Microbial biomass C (g kg^–1^)	Succrase activity (mg g^–1^ day^–1^)	Urease activity (mg g^–1^day^–1^)	Cellulase activity (mg g^–1^day^–1^)	Protease activity (μg g^–1^day^–1^)
Transplanting (TP)	RS	158.7	1.7 d	8.4 a	7.5 d	1.2 b	143.2 a
	BC	167.4	1.7 c	8.1 b	9.5 a	1.0 d	124.5 c
	CK	161.0	1.6 f	7.4 d	7.3 de	1.0 d	124.9 c
Direct seeding (DS)	RS	168.4	1.8 b	7.6 c	7.1 e	1.3 a	132.9 b
	BC	165.2	1.9 a	7.6 c	9.0 b	1.2 b	115.8 d
	CK	162.3	1.6 e	7.2 e	7.7 c	1.1 c	106.9 e
	LSD_(0.05)_	1.55	<0.01	0.07	0.20	0.02	2.20
Planting methods							
TP		162.3 b	1.67 b	7.9 a	8.1	1.1 b	130.9 a
DS		165.3 a	1.77 a	7.5 b	8.0	1.2 a	118.6 b
Organic amendments							
RS		163.5 b	1.75 b	8.0 a	7.3 b	1.23 a	138.1 a
BC		166.3 a	1.80 a	7.9 b	9.3 a	1.10 b	120.2 b
CK		161.6 b	1.60 c	7.3 c	7.5 c	1.05 c	115.9 c
Analysis of variance							
Planting methods (PM)		**	**	*	NS	**	*
Organic amendments (OA)		**	**	**	**	**	**
PM × OA		**	**	**	**	*	*

Different letters indicate statistical significance at the P < 5% level within planting methods application methods. *Significant at 0.05 level; **Significant at 0.01 level; NS, Not significant at 0.05 level. RS, rice straw; BC, biochar; CK, no RS or BC.

### Soil physicochemical properties

There were significant differences in soil pH, organic C (OC), available N (AN), available P (AP), available K (AK), NO_3_^–^-N (NON), and NH_4_^+^-N (NHN) between the different seeding methods and among organic amendments (*P* < 0.05) (Table 3). Specifically, OC and NHN were significantly higher for TP relative to DS, while the opposite trend was observed for AP and AK. RS amendment was associated with increases in OC, AP, NON, and NHN contents relative to BC and CK amendment conditions. BC application led to increases in pH, AN, and AK relative to RS and CK. RS application was associated with increased rhizosphere soil NHN and OC for TP and with increased rhizosphere soil AP for DS, while BC application improved rhizosphere soil AK contents.

**TABLE 3:** Soil physicochemical characteristics under different treatments of planting methods and organic amendments.

Planting methods	Organic amendments	pH	Organic C (g kg^–1^)	Available N (mg kg^–1^)	Available P (mg kg^–1^)	Available K (mg kg^–1^)	NO_3_^–^-N (mg kg^–1^)	NH_4_^+^-N (mg kg^–1^)
Transplanting (TP)	RS	5.7	13.3 a	121.5 b	28.6 a	104.3c	1.43b	3.1 a
	BC	5.9	13.2 a	120.4 b	27.1 b	123.0 a	1.33c	3.0 a
	CK	5.5	11.9 d	115.7 c	25.0 c	98.9 d	1.2 e	2.5 d
Direct seeding (DS)	RS	5.8	12.3 c	125.0 a	27.5 b	115.2 b	1.5 a	2.9 b
	BC	5.9	13.0 b	120.8 b	28.5 a	122.7 a	1.3 c	2.7 c
	CK	5.6	12.0 d	110.5 d	25.4 c	105.6 c	1.3 d	2.0 e
	LSD_(0.05)_	0.04	0.08	0.7	0.5	2.3	0.02	0.05
Planting methods								
TP		5.7	12.8 a	119.2	26.9 b	108.7 b	1.3	2.9 a
DS		5.7	12.4 b	118.8	27.1 a	114.5 a	1.4	2.5 b
Organic amendments								
RS		5.7 b	12.8 b	123.2 a	28.1 a	109.7 b	1.47 a	2.97 a
BC		5.9 a	13.1 a	120.6 b	27.8 b	122.9 a	1.32 b	2.83 b
CK		5.6 c	11.9 c	113.1 c	25.2 c	102.3 c	1.25 c	2.28 c
Analysis of variance								
Planting methods (PM)		NS	**	NS	*	*	NS	240.3**
Organic amendments (OA)		**	**	**	**	**	**	257.6**
PM × OA		NS	**	**	*	*	**	11.1**

Different letters indicate statistical significance at the P < 5% level within planting methods application methods. *Significant at 0.05 level; **Significant at 0.01 level; NS, Not significant at 0.05 level. RS, rice straw, BC, biochar, and CK, no RS or BC.

### Relationships between bacterial community structure and enzymatic, physiochemical properties, and yield

**FIGURE 5:**
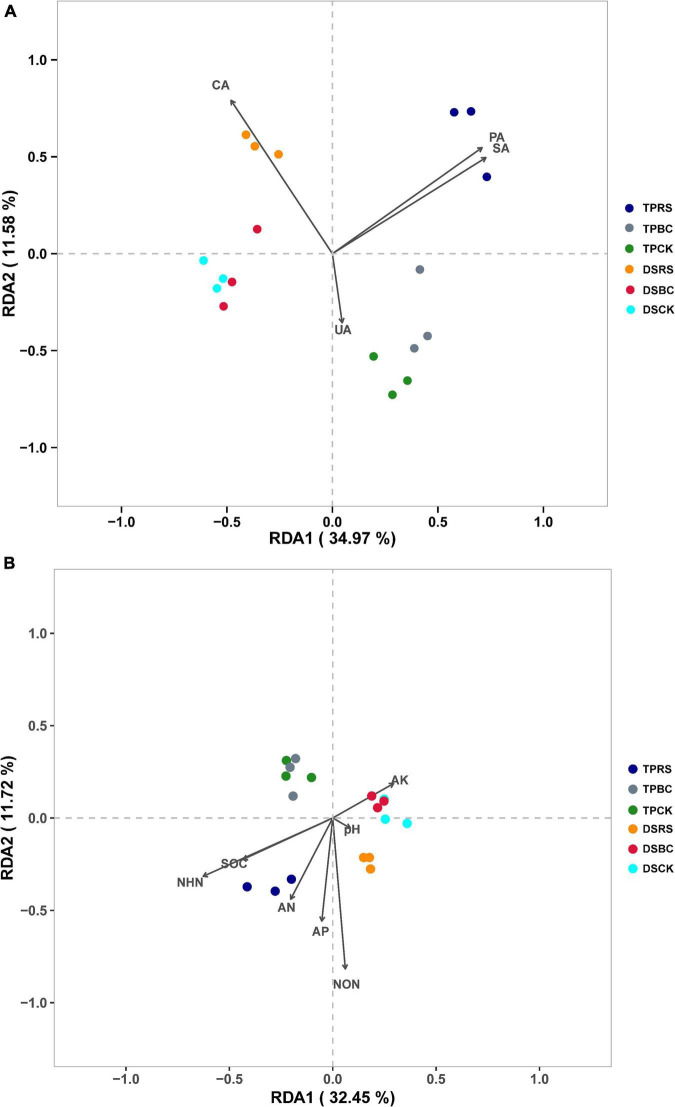
**(A,B)** Redundancy analysis of rhizosphere soil bacterial genera and soil enzyme activity and physicochemical characteristics.

RDA ordination plots were further used to explore relationships among soil bacterial communities, soil physicochemical properties, and soil enzyme activity levels. Both planting methods and organic amendments were associated with distinct impacts on soil bacterial communities under these tested conditions (Figure 5). While rhizosphere soil bacteria associated with the CK and BC amendments were similar to one another under both planting methods, they were clearly separated from those bacterial communities associated with the RS. Separation was also evident between bacterial communities associated with DS and TP. SA, PA, AN, AP, SOC, NON, and NHN were significantly correlated with soil bacterial community composition under conditions of RS amendment, while CA and NON were significantly correlated with soil bacterial communities when RS was applied under DS (Figure 5).

### Relationships among abundant phyla, enzyme activities, and soil physiochemical properties

Pearson’s correlation analyzes were used to examine relationships among rhizosphere bacterial phyla abundance, soil properties, and soil enzyme activity levels (Tables 4–6). *Bacteroidetes* abundance was positively correlated with SA, PA, SOC, NHN, full grains per panicle and yield. *Chloroflexi* abundance was negatively correlated with CA and NON, but positively correlated with soil SA, NHN, full grains per panicle and yield. *Gemmatimonadetes* abundance was positively correlated with CA and number of panicles, but negatively correlated with full grains per panicle and yield. *Nitrospirae* abundance was positively correlated with SA, PA, NHN and yield. *Patescibacteria* abundance was negatively correlated with CA, PA and NON, but positively correlated with soil UA. *Proteobacteria* abundance was positively correlated with CA, number of panicles.and 1000 seed weight. *Verrucomicrobia* abundance was negatively correlated with UA, PA, SOC, AN, AP, NHN and yield. The Supplementary Tables 1, 2 showed that SA was positively correlated with full grains per panicle and yield, but negatively correlated with number of panicles. Protease activity and NHN were positively correlated with full grains per panicle and yield. Cellulose activity was positively correlated with number of panicles and 1000 seed weight. The Supplementary Table 3 showed that SA was positively correlated with OC, AN, AP, NON and NHN, and UA was positively correlated with pH, OC and AK. Cellulose activity was positively correlated with pH, AN, AP and NON. Protease activity was positively correlated with AN, AP, NON and NHN.

**TABLE 4:** Pearson’s correlation coefficients between abundant phyla, microbial biomass and enzyme activities.

	Succrase activity	Urease activity	Cellulase activity	Protease activity
*Bacteroidetes*	0.77**	0.1	–0.41	0.70**
*Chloroflexi*	0.49*	0.18	–0.58*	0.47
*Gemmatimonadetes*	–0.46	–0.29	0.61**	–0.32
*Nitrospirae*	0.53*	–0.28	0.03	0.75**
*Patescibacteria*	–0.37	0.54*	–0.56*	–0.54*
*Proteobacteria*	–0.04	–0.13	0.71**	–0.10
*Verrucomicrobia*	–0.62**	0.12	–0.19	–0.65**

Values are indicate statistically significant.

*P < 0.05; **P < 0.01

**TABLE 5:** Pearson’s correlation coefficients between abundant phyla and soil physicochemical properties.

	pH	Organic C	Available N	Available P	Available K	NO_3_^–^-N	NH_4_^+^-N
*Bacteroidetes*	0.03	0.55*	0.24	0.18	–0.2	0.06	0.66**
*Chloroflexi*	0.01	0.32	0.15	–0.07	–0.13	–0.16	0.53*
*Gemmatimonadetes*	–0.05	–0.37	–0.07	0.03	0.12	0.3	–0.44
*Nitrospirae*	–0.04	0.23	0.43	0.21	–0.26	0.41	0.58*
*Patescibacteria*	0.12	–0.13	–0.28	–0.37	0.36	–0.64**	–0.24
*Proteobacteria*	0.21	0.07	0.1	0.39	0.15	0.42	–0.16
*Verrucomicrobia*	–0.25	–0.47*	–0.51*	–0.47*	–0.02	–0.44	–0.62**

Values in italics are indicate statistically significant.

*P < 0.05; **P < 0.01.

**TABLE 6:** Pearson’s correlation coefficients between abundant phyla and grain yield and components.

	Number of panicles	Full grains per panicle	Percentage of filled grains	1000 seed weight	Yield
*Bacteroidetes*	–0.88**	0.92**	–0.06	–0.35	0.83**
*Chloroflexi*	–0.82**	0.82**	–0.03	–0.53*	0.66**
*Gemmatimonadetes*	0.75**	–0.75**	–0.05	0.47	–0.57*
*Nitrospirae*	–0.49*	0.52*	–0.22	–0.16	0.60**
*Patescibacteria*	0.01	–0.06	0.2	–0.38	–0.28
*Proteobacteria*	0.59*	–0.55*	–0.06	0.54*	–0.36
*Verrucomicrobia*	0.33	–0.45	–0.04	–0.16	–0.58*

Values in italics are indicate statistically significant.

*P < 0.05; **P < 0.01.

### Discussion

DS and TP are the two primary approaches to paddy rice planting. Incorporation of both RS and BC has shown to be a practical approach to enhancing soil quality and productivity (Zhao et al., 2014a; Wang et al., 2015). Crop straw return in the present study was associated with increases in grain yields (Table 1), in line with prior evidence that RS, combined with the surface application of chemical fertilizers, can significantly enhance crop yields (Zhao et al., 2017; Kumari et al., 2018). However, these results are not universal, with some researchers reporting an absence of grain yield increase in the context of crop straw return and fertilizer application (Sun et al., 2018). In this study, RS significantly increased grain yields concerning the number of panicles and the number of full grains per panicle. In prior reports, TP was shown to be necessary to achieve reliably high crop yields with respect to an increase in the spikelet number per panicle, consistent with an increase in the number of filled grains per panicle relative to DS (Kamiji et al., 2011; Xing et al., 2017). These results are consistent with our findings. The observed increase in grain yields for the TP relative to DS was primarily attributable to the rise in the number of filled grains per panicle. With TP, the sowing date was earlier and the duration before heading was longer than that with DS, which were conducive to improve dry matter of crops, resulting in a larger panicle and improving grain yield (Chen et al., 2009). Compared to DS, SA, PA and NHN improved with TP (Tables 2,3), and SA, PA and NHN were positively correlated with full grains per panicle (Supplementary Tables 1, 2). Transplanting had more filled grains per panicle which may be an essential factor for improving grain yield.

The positive impact of RS and BC amendments in the present study is mainly likely attributable in large part to improvements in soil physicochemical properties. For instance, RS amendment was associated with increases in OC, AP, NON, and NHN contents and BC application increased pH, AN, and AK (Ding et al., 2016; Blanco-Canqui, 2017; Chen et al., 2017). Indeed, improvements in several soil properties were observed following RS or BC amendment relative to CK, in line with prior reports (Chen et al., 2017; Andrey et al., 2020). Specifically, RS application was associated with significant increases in paddy soil OC, AP, NON and NHN, potentially owing to the enhanced decomposition of RS by enzymes and microbes within the soil, leading to enhanced straw nutrient release into the soil. Biochar was also improved soil pH, AN and AK contents. Prior reports suggest that BC application can increase the pH of acidic soils and reduce nutrient loss through the enhancement of water retention (Joseph et al., 2010), consistent with our results.

The activity of soil enzymes can offer valuable insight regarding nutrient cycling (Nannipieri et al., 2003; Aon and Colaneri, 2011), as these activity levels regulate rates of nutrient cycling and organic matter decomposition in the soil (Nannipieri et al., 2012). In this study, both planting methods and organic amendments were associated with altered rhizosphere soil enzyme activity and microbial biomass (Table 2). Significant improvements in soil SA and PA were observed for TP, which may be owing to the high content of NHN, that was positively correlated with soil SA and PA. In contrast, DS was associated with improved rhizosphere soil MBN, MBC and CA, because of the higher AK content in DS (Supplementary Table 3). The RS application improved the rhizosphere soil AN, AP, NON, and NHN. The AN, AP, NON and NHN were positively correlated with SA, CA and PA (Supplementary Table 3), The application of RS increased SA, CA, and PA. Because of the addition of readily degraded substrate RS contributed to increases in sucrase, cellulase, and protease activity, in line with prior reports (Sardans et al., 2008; Wu et al., 2013; Wei et al., 2015; Zhu et al., 2017; Bao et al., 2020). These soil enzymes and associated microbes break RS down into cellulose and hemicelluloses that are then further degraded to yield carbohydrates, starches, polysaccharides, and simple sugars (Hansen et al., 2016). Soil enzymes and microbes associated with the RS-amended soil are more efficient in carbohydrate substrate utilization. In contrast, the porous structure of BC is associated with enhanced N and C fixing in the soil, enabling certain beneficial species of microbes to thrive (Hagemann et al., 2017). This is likely the primary explanation for the observed increases in UA and soil MBC and MBN in BC-amended soil relative to those observed in CK or RS amendment conditions.

Sustainable and productive cropping systems necessitate the maintenance of pronounced microbial diversity and richness (Zhao et al., 2014b). A loss of such diversity often occurs in the context of prolonged agricultural cultivation, while RS and BC amendments can positively impact both bacterial diversity and abundance (Sun et al., 2016; Chen et al., 2017), consistent with the present results.

The abundance of *Chloroflexi*, *Bacteroidetes*, and *Nitrospirae* increased in the RS-amended TP (Figure 4). In TP, RS amendment improved soil NHN, SA and PA, whereas the *Chloroflexi*, *Bacteroidetes* and *Nitrospirae* abundance were positively correlated with SA, PA, NHN, and grain yield (Tables 4–6). The increased relative abundance of these microbial in the soil is beneficial for improving grain yield. *Gemmatimonadetes* abundance was significantly increased in the RS-amended DS. *Gemmatimonadetes*, highly abundant phyla associated with RS amendment, have been shown to reduce cellulose metabolic byproducts, thus indirectly facilitating the degradation of cellulose (Takaichi et al., 2010). *Gemmatimonadetes* abundance was positively correlated with CA, but negatively correlated with full grains per panicle and yield. This may be the reason why the grain yield of DS was lower than TP.

The RDA ordination plots indicated that both seeding methods and organic amendments had an impact on the structure and rhizosphere soil bacterial communities (Figure 5). Zhao et al. (2017) found that straw addition was associated with pronounced separation from control soil bacterial communities, with the composition of these communities significantly correlated with soil physicochemical properties. In the present study, the soil bacterial communities associated with the CK and BC amendment conditions were similar and clearly separated from those associated with RS amendment for both DS and TP. These data suggest that RS can markedly influence rhizosphere soil bacterial communities. Under conditions of RS amendment, SA, CA, PA, AN, AP, NON, and NHN were significantly correlated with bacterial communities, owing to the participation of many microbes and soil enzymes in the context of straw decomposition and nutrient consumption, in line with prior reports (Zhao et al., 2017; Chen et al., 2020). Under conditions of BC amendment, UA and pH were significantly correlated with bacterial communities (Figure 5). This may be attributable to the porous structure of BC, resulting in soil nutrient immobilization, or to its alkalinity. RDA ordination plots indicated that all three organic amendments were separated from one another under both DS and TP. For DS, the original plow layer was destroyed and the water layer could not be maintained throughout the entirety of the rice-growing period. The consequent alternating wetting and drying of the soil may have contributed to changes in rhizosphere soil microorganisms.

Both RS and BC additions affected rhizosphere soil nutrient concentrations, enzymatic activity, microbial diversity, and abundance. Copitrophic bacteria thrive when resource levels are abundant, whereas oligotrophs exhibit greater relative abundance when resource levels are limited (Eilers et al., 2010). The results of the present study revealed a positive correlation between *Proteobacteria* abundance and CA, while *Gemmatimonadetes* abundance was positively correlated with CA. In addition, *Nitrospirae* abundance was positively correlated with SA, PA and NHN, whereas *Bacteroidetes* abundance was positively correlated with SA, PA, OC and NHN. These data align well with prior reports (Ramirez et al., 2012), suggesting that RS and BC can increase local nutrient contents, thereby preferentially promoting the growth of bacteria best suited to nutrient-rich environments, including *Proteobacteria* and *Nitrospirae*. These data thus support the ability of organic amendments to promote the growth of copiotrophic bacteria, which has the potential to enhance the productivity and sustainability of soil under intensive agricultural systems.

## Conclusion

Our study proved that both planting methods and organic amendments altered the abundance and diversity of bacterial communities in the rhizosphere soil. RS application could enhance yields of paddy rice under DS and TP. Moreover, both RS and BC applications were sufficient to improve soil nutrient availability and enzymatic activity under DS and TP. RS application thus significantly influenced soil microbial community structure, promoted the preferential growth of copiotrophic bacteria, and increased grain yield. This study marks a crucial step toward a more in-depth knowledge of the mechanisms by which planting methods and organic amendments influence the composition and variety of rhizosphere soil microbial communities, soil enzyme activity levels and soil physicochemical attributes.

## Data availability statement

The datasets presented in this study can be found in online repositories. The names of the repository/repositories and accession number(s) can be found below: SUB11374070, PRJNA833904.

## Author contributions

ZT contributed significantly to the field experiment, data collection and analysis, and wrote the original manuscript. LZ, NH, ZM, LF, HW, and CW performed the field experiment. WZ and GS helped to perform the analysis with constructive discussions and contributed to the conception of the study. All authors contributed to the article and approved the submitted version.
