# Supplementary material for: Influence of planting methods and organic amendments on rice yield and bacterial communities in the rhizosphere soil
Source: Front Microbiol. 2022 Jul 28;13:918986. doi: 10.3389/fmicb.2022.918986 (PMC9366738; doi:10.3389/fmicb.2022.918986)
Supplement: Supplementary file 1 [file Data_Sheet_1.PDF]

Supplementary Material

1. Supplementary Tables

Table S1. Pearson's correlation coefficients between enzyme activities and grain yield and components.

	Number of panicles	Full grains per panicle	Percentage of filled grains	1000 seed weight	Yield
Sucrase	-0.57*	0.67**	-0.07	-0.06	0.76**
Urease	-0.13	0.06	0.12	-0.48*	-0.18
Cellulase	0.69**	-0.35	-0.21	0.95**	-0.18
Protease	-0.46*	0.62**	-0.06	0.23	0.87**

*P < 0.05 and **P < 0.01

Table S2. Pearson's correlation coefficients between soil properties and grain yield and components.

	Number of panicles	Full grains per panicle	Percentage of filled grains	1000 seed weight	Yield
pH	0.22	-0.16	-0.10	0.05	-0.10
Organic C	-0.38	0.43	-0.16	-0.14	0.43
Available N	0.03	0.13	-0.01	0.38	0.43
Available P	0.05	0.05	-0.35	0.38	0.24
Available K	0.32	-0.29	0.02	0.01	-0.27
NO_3^--N	0.31	-0.13	-0.30	0.62**	0.24
NH_4^+-N	-0.43	0.57*	0.04	0.01	0.75**

*P < 0.05 and **P < 0.01

TableS3. Pearson's correlation coefficients between soil properties and enzyme activities.

	pH	Organic C	Available N	Available P	Available K	NO_3^--N	NH_4^+-N
Sucrase	0.44	0.88**	0.62**	0.70**	0.25	0.54*	0.87**
Urease	0.75**	0.62**	0.08	0.28	0.76**	-0.18	0.21
Cellulase	0.56*	0.04	0.55*	0.58**	0.26	0.49*	0.15
Protease	0.06	0.43	0.70**	0.51*	-0.16	0.54*	0.81**

*P < 0.05 and **P < 0.01

2.Supplementary Figures

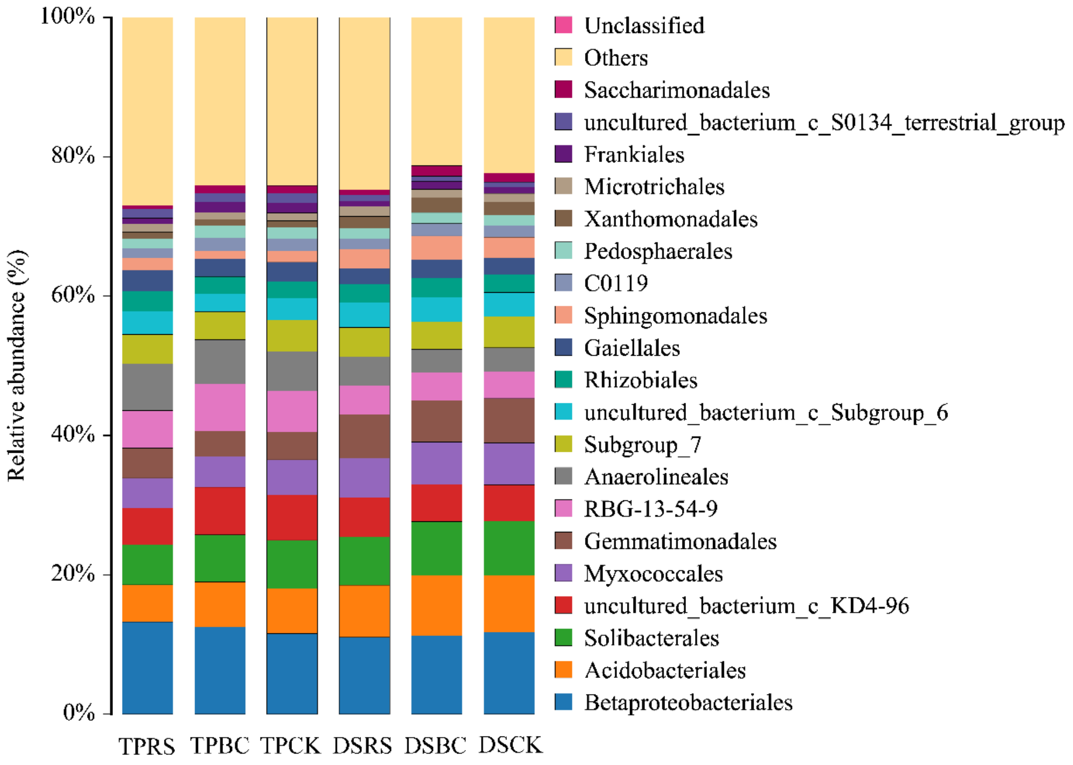

Figure S1. Order level bacterial community composition following the rhizosphere treatments. Only orders with more than 1% relative abundance are shown. TP = transplanting, DS = direct seeding, RS = rice straw, BC = biochar, and CK = no RS or BC.

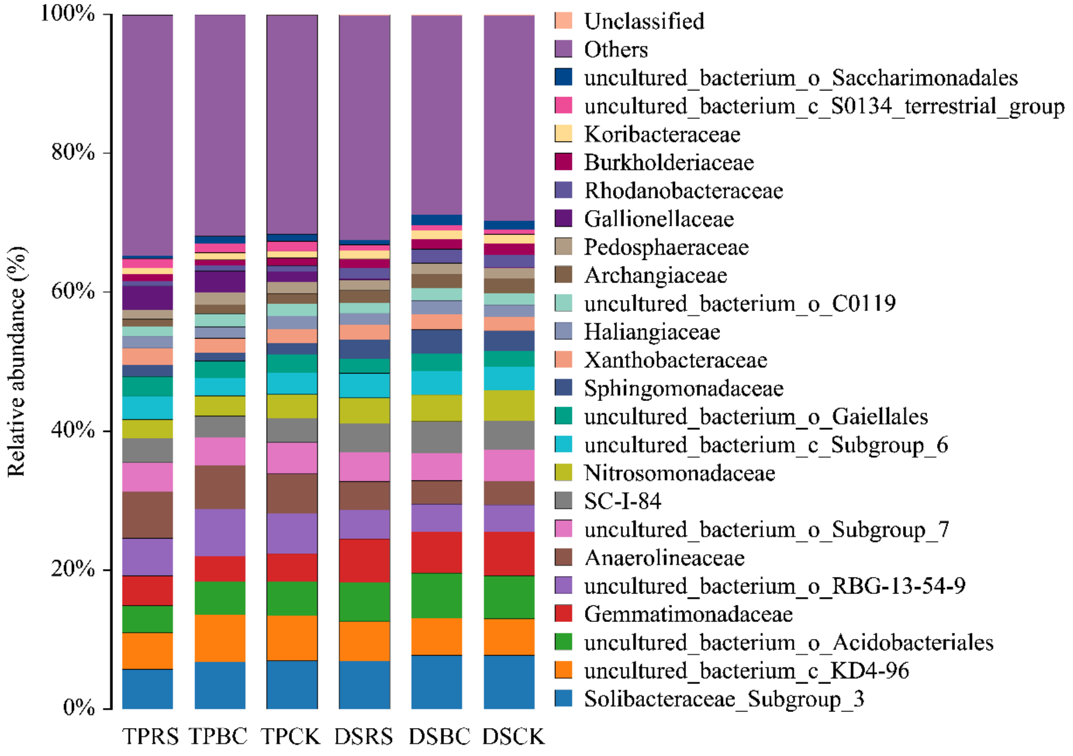

Figure S2. Family level bacterial community composition following the rhizosphere treatments. Only families with more than 1% relative abundance are shown. TP = transplanting, DS = direct seeding, RS = rice straw, BC = biochar, and CK = no RS or BC.

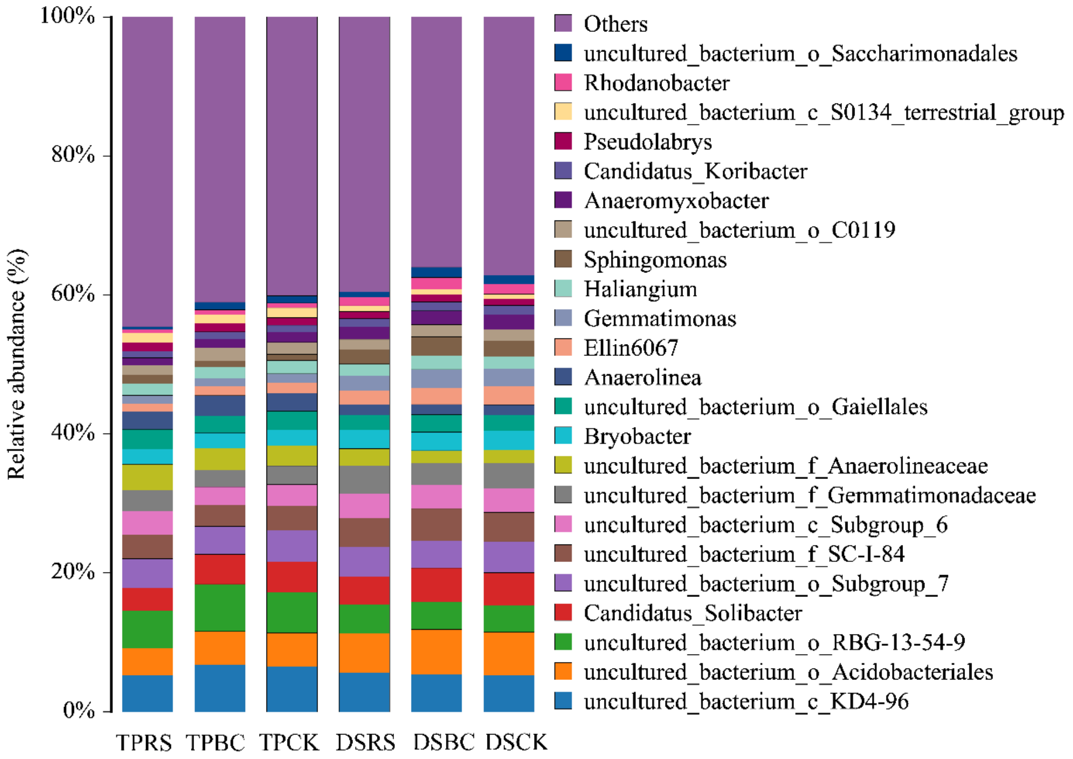

Figure S3. Genus level bacterial community composition following the rhizosphere treatments. Only genus with more than 1% relative abundance are shown. TP = transplanting, DS = direct seeding, RS = rice straw, BC = biochar, and CK = no RS or BC.
